# Insights Into the Spatiotemporal Patterns of Complexity of Ventricular Fibrillation by Multilead Analysis of Body Surface Potential Maps

**DOI:** 10.3389/fphys.2020.554838

**Published:** 2020-09-23

**Authors:** Marianna Meo, Arnaud Denis, Frédéric Sacher, Josselin Duchâteau, Ghassen Cheniti, Stéphane Puyo, Laura Bear, Pierre Jaïs, Mélèze Hocini, Michel Haïssaguerre, Olivier Bernus, Rémi Dubois

**Affiliations:** ^1^Institute of Electrophysiology and Heart Modeling (IHU Liryc), Foundation Bordeaux University, Bordeaux, France; ^2^Centre de Recherche Cardio-Thoracique de Bordeaux, U1045, University of Bordeaux, Bordeaux, France; ^3^Centre de Recherche Cardio-Thoracique de Bordeaux, U1045, Institut National de la Santé et de la Recherche Médicale, Bordeaux, France; ^4^Electrophysiology and Ablation Unit, Bordeaux University Hospital, Bordeaux, France

**Keywords:** ventricular fibrillation, complexity, body surface potential maps, singular value decomposition, sudden cardiac death, electrocardiology, structural diseases, ventricular fibrillation mechanisms

## Abstract

**Background:**

Ventricular fibrillation (VF) is the main cause of sudden cardiac death, but its mechanisms are still unclear. We propose a noninvasive approach to describe the progression of VF complexity from body surface potential maps (BSPMs).

**Methods:**

We mapped 252 VF episodes (16 ± 10 s) with a 252-electrode vest in 110 patients (89 male, 47 ± 18 years): 50 terminated spontaneously, otherwise by electrical cardioversion (DCC). Changes in complexity were assessed between the onset (“VF start”) and the end (“VF end”) of VF by the nondipolar component index (*NDI*_*BSPM*_), measuring the fraction of energy nonpreserved by an equivalent 3D dipole from BSPMs. Higher NDI reflected lower VF organization. We also examined other standard body surface markers of VF dynamics, including fibrillatory wave amplitude (*A*_*BSPM*_), surface cycle length (*BsCL*_*BSPM*_) and Shannon entropy (*ShEn*_*BSPM*_). Differences between patients with and without structural heart diseases (SHD, 32 vs. NSHD, 78) were also tested at those stages. Electrocardiographic features were validated with simultaneous endocardium cycle length (CL) in a subset of 30 patients.

**Results:**

All BSPM markers measure an increase in electrical complexity during VF (*p* < 0.0001), and more significantly in NSHD patients. Complexity is significantly higher at the end of sustained VF episodes requiring DCC. Intraepisode intracardiac CL shortening (VF start 197 ± 24 vs. VF end 169 ± 20 ms; *p* < 0.0001) correlates with an increase in NDI, and decline in surface CL, f-wave amplitude, and entropy (*p* < 0.0001). In SHD patients VF is initially more complex than in NSHD patients (*NDI*_*BSPM*_, *p* = 0.0007; *ShEn*_*BSPM*_, *p* < 0.0001), with moderately slower (*BsCL*_*BSPM*_, *p* = 0.06), low-amplitude f-waves (*A*_*BSPM*_, *p* < 0.0001). In this population, lower NDI (*p* = 0.004) and slower surface CL (*p* = 0.008) at early stage of VF predict self-termination. In the NSHD group, a more abrupt increase in VF complexity is quantified by all BSPM parameters during sustained VF (*p* < 0.0001), whereas arrhythmia evolution is stable during self-terminating episodes, hinting at additional mechanisms driving VF dynamics.

**Conclusion:**

Multilead BSPM analysis underlines distinct degrees of VF complexity based on substrate characteristics.

## Introduction

Sudden cardiac death is a major health problem worldwide accounting for 8% to 12% of all deaths (Hayashi et al., 2015), with global annual incidence rates between 50 and 100 per 100,000 cases (Fishman et al., 2010).

Ventricular fibrillation is the most serious heart rhythm disturbance and the main cause of SCD (Priori et al., 2015). Early studies interpreted the apparently chaotic and random activation of the myocardium during VF as totally disorganized (Moe et al., 1964). Later, the development of multielectrode (Rogers et al., 1999) and optical mapping (Park and Gray, 2015) techniques for the analysis of the electrical activation of the myocardium has enabled a more detailed evaluation of the electrical substrate of the arrhythmia, and both multiple wavelet and mother rotor hypotheses have been thought to be involved in VF initiation and maintenance (Jalife, 2000; Weiss et al., 2005). However, despite such advances, the mechanisms underlying this electrical disorder are still controversial. Yet, a quantitative assessment of VF organization may help understanding which factors determine the onset, perpetuation, and cessation of this arrhythmia (Such-Miquel et al., 2013), and be clinically relevant in the outline of more effective strategies for defibrillation and resuscitation, and the prediction of shock timings and success.

There is an increasing interest in BSPM technologies to noninvasively measure cardiac potentials with higher spatial resolution compared with standard 12-lead ECG. Indeed, subtle anomalies underlying VF may be missed by current imaging technologies, and rather bring a specific electrical signature, which may be critical in the treatment of patients with structurally normal hearts (Cheniti et al., 2018). However, a few studies have attempted a detailed analysis of the complexity of VF from multiple electrodes on body surface, due to the impossibility to quickly attach large sets of leads to a patient and acquire intraepisode recordings (Fitz-Clarke et al., 2006). Accordingly, most of the metrics currently used to describe VF from ECG are mainly determined from isolated leads, e.g., dominant frequency (Eftestøl et al., 2000) or entropy (Chicote et al., 2016), and a few multilead approaches have been developed (Clayton et al., 1995; Fitz-Clarke et al., 2006). Furthermore, to our knowledge, none of these works has clearly elucidated whether and how surface electrical patterns of VF may reflect underlying mechanisms.

Motivated by all these elements, this study aims put forward a multilead methodology to noninvasively describe spatiotemporal progression of VF and investigate how complexity patterns evolve in the presence/absence of SHDs.

## Materials and Methods

### VF Population

This study included 110 patients (89 male, 47 ± 18 years) enrolled for VF ablation. As in Haïssaguerre et al. (2018), SHDs were screened by echocardiography and delayed gadolinium-enhanced magnetic resonance imaging in 32 patients, whereas the remaining subjects (78) presented no structural alterations (NSHD). Pharmacological testing was performed by injecting ajmaline or catecholamine (adrenaline and isoprenaline) to diagnose patients with Brugada syndrome, long QT, and catecholaminergic polymorphic VT, respectively. This study was carried out in accordance with the recommendations of the protocol CARRY, ID-RCB: 2015-A00401-48, *Comité de Protection des Personnes Sud-Ouest et Outre Mer III* (ClinicalTrial.gov number: NCT02647749). It was approved by the Institutional Clinical Research and Ethics Committee and all subjects gave written informed consent in accordance with the Declaration of Helsinki. Baseline information for this patients’ cohort are detailed in Table 1.

**TABLE 1:** Clinical characteristics of the VF population.

	**SHD (*n* = 32)**	**NSHD (*n* = 78)**	***p*-value**
Sex, male (*n*)	26	63	**<0.0001**
Age, mean ± std (years)	58 ± 18	41 ± 18	**<0.0001**
Pathology subtype (*n*) (no. male)	Ischemic VF 15 (13)	Brugada syndrome 29 (28)	
	HCM 9 (7)	Idiopathic VF 25 (17)	
	DCM 3 (2)	ERS 8 (6)	
	VT 3 (2)	ERP 6 (6)	
	OHCM 1 (1)	Brugada syndrome and ERP 4 (4)	
	LVD 1 (1)	Torsade de pointe 4 (1)	
		Long QT syndrome 1 (0)	
		Catecholaminergic VT 1 (1)	

*DCM, dilated cardiomyopathy; ERP, early repolarization pattern; ERS, early repolarization syndrome; HCM, hypertrophic cardiomyopathy; LVD, left ventricular dysfunction; OHCM, obstructive hypertrophic cardiomyopathy; std, standard deviation; VT, ventricular tachycardia; *p-*values in boldface are statistically significant vs. “SHD”. For each pathology subtype, the number of male patients is reported between brackets.*

### Noninvasive Mapping of VF and BSPM Preprocessing

A mean of 2.3 ± 2.1 spontaneous or induced VF episodes/patient were mapped with a 252-electrode vest (CardioInsight, Medtronic, MN, United States) at a sampling rate of 1 kHz before ablation, with a total of 177 episodes recorded in the NSHD group, and 75 episodes from the SHD group. Mean duration of VF was 18 ± 10 s. In keeping with current guidelines (Al-Khatib et al., 2017), VF was defined as rapid, grossly irregular electrical activity with marked variability in electrocardiographic waveform, and cycle length (CL) < 200 ms. VF rhythms have been reviewed and annotated by expert electrophysiologists. As in Haïssaguerre et al. (2018), the duration of VF episodes was dependent on the time-to-charge of the external defibrillator before shock delivery. The longer this waiting time, the longer the duration of the recorded episode, which was then labeled as “Sustained VF” if shock was delivered. Conversely, episodes with spontaneous reversion of VF to sinus rhythm before the cardioversion (DCC) discharge were referred to as “self-terminating VF.” Baseline wander was suppressed by the median estimation method (Sörnmo and Laguna, 2005). Body surface potentials were arranged as a *L*×*N* matrix **Y** = [**y**(1)…**y**(*N*)] ∈ ℜ^*L*×*N*^, where *L* = 252 is the number of BSPM electrodes, and *N* the number of time samples. Signal quality was assessed by visual inspection, and too noisy electrode recordings were discarded if required, therefore in some patients we may have *L* < 252.

### Electrophysiological Study and Ablation

Electroanatomical mapping (CARTO system, Biosense Webster, CA, United States) was performed as in Haïssaguerre et al. (2018). Briefly, a transseptal or retroaortic approach was adopted to access the endocardial left ventricle and a subxyphosternal approach to access into the pericardial space. Decapolar and multispline (Pentaray, Biosense Webster, CA, United States) catheters were used for endocardial and epicardial exploration, respectively. Intracardiac electrograms were recorded and stored by a computer-based digital amplifier/recorder system (Labsystem Pro, Bard Electrophysiology). VF was induced by programmed stimulation from one of the ventricles (mainly the right one), if required. Endocardial VF CLs were measured mainly in the right ventricle. After mapping, VF ablation was subsequently performed with an irrigated-tip catheter as in Haïssaguerre et al. (2018) and targeted ventricular sites with abnormal electrograms and driver activities estimated by ECG imaging.

### Novel Multilead Methodology to Measure VF Complexity

Previous research has shown that ventricular repolarization could be effectively described in an orthogonal 3D space and that higher-order components reflected T-wave spatial heterogeneities, representing an arrhythmogenic substrate (De Ambroggi et al., 1997; Priori et al., 1997; Malik et al., 2000). Similar approaches have also been applied to the quantification of QRS dispersion and prediction of sudden death (Peters et al., 1999; Turrini et al., 2001). Moving from the theoretical basis in Di Marco et al. (2012), Meo et al. (2017) and Meo et al. (2018), we measured spatiotemporal organization of VF using the nondipolar component index (NDI), namely, the fraction of signal energy that is not captured by a 3D dipolar approximation of body surface cardiac activity. However, unlike those studies, we were more interested in determining the temporal evolution of VF complexity rather than its static characteristics. Accordingly, the input BSPMs were divided in 500-ms windows. Each multilead signal frame was then mean-centered and projected on a 3D subspace estimated by SVD:

$${\text{Y}}^{\left(s\right)}\,=\,{\text{USV}}^{\text{T}}$$

With **U** and **V** standing for the left and right singular vectors of **Y**^(*s*)^, respectively, and the diagonal matrix **S** containing the singular values σ_ℓ_, ℓ = 1,…,*L*. In line with the mathematical formulation proposed by Meo et al. (2018), NDI was computed as:

$${\text{NDI}}_{B\,S\,P\,M}\,=\,100\cdot \left(1-\frac{\sum_{\ell \,=\,1}^{3}\sigma_{\ell }^{2}}{\sum_{\ell \,=\,1}^{L}\sigma_{\ell }^{2}}\right)$$

under the hypothesis that more organized VF patterns could be properly described by a 3D cardiac dipole and yield lower NDI values, whereas more variable and unpredictable waveforms (both in time and across leads) would be less likely to be accurately described by this model, thus they will be associated with higher NDI values. With regard to the choice of the frame duration, the proposed value was selected as a trade-off between the amount of input information and SVD applicability, as we needed to accurately capture spatiotemporal variability properties of signal patterns from least two to three fibrillatory cycles, while preserving data stationarity within the BSPM frame and obtaining reliable measures (Meo et al., 2018). Since endocardial CL in our validation subset ranged between 150 and 250 ms, our choice proved to be suitable. The effect of tuning parameters on SVD-derived features has been more systematically tested in previous works (Meo et al., 2013a).

### Other Body Surface Markers of VF Electrical Complexity and Dynamics

Organization of VF and its time evolution have also been assessed by other signal parameters reported in literature and conventionally computed from single-lead ECG.

Fibrillatory wave (f-wave) is known to be predictive of successful defibrillation shocks (Strohmenger et al., 2001) in patients with cardiac arrest, and its decline in time reflects increasing intracellular electrical decoupling with VF progression (Caldwell et al., 2012). Accordingly, this parameter was computed from each electrode using a custom approach based on the interpolation of signal local extrema through polynomial envelopes (Meo et al., 2013b, 2018). The output amplitude index was the median value over all electrodes and denoted *A*_*BSPM*_.

The same method also served as a preliminary step for the assessment of body surface VFCL, as faster activations typically correlate with duration of VF and are less likely to be terminated by defibrillation (Strohmenger et al., 2001). As in Meo et al. (2018), signal local maxima were detected by a derivative-threshold algorithm, and the median of CLs between 90 and 250 ms was computed over all BSPM leads (body surface VFCL, denoted as *BsCL*_*BSPM*_), to reject the influence of false peaks.

Finally, complexity of VF has also been quantified by a nonlinear index, i.e., Shannon entropy, under the hypothesis that less regular and repetitive patterns are characterized higher uncertainty in a statistical sense. We assume that lower values of the index render more predictable times series. Entropy-based indices are widely used to detect VF rhythms (Hajeb-Mohammadalipour et al., 2018) and predict defibrillation outcome (Chicote et al., 2016). The ability of this parameter to identify rotational activities in atrial fibrillation patients has also been demonstrated (Ganesan et al., 2013). To account for contributions from all BSPM recordings, Shannon entropy has been determined with histogram bin width set at 0.01 mV, and the median *ShEn*_*BSPM*_ over all electrodes has been assumed as a marker for VF complexity.

We also investigated the scalability of BSPM-derived features to 12-lead ECG for a potential application of VF complexity analysis in a larger clinical scenario. For this purpose, each of the aforementioned parameters has been also computed from an equivalent ECG obtained from a subset of BSPM electrodes at the locations of standard leads and denoted *NDI*_*ECG12*_, *A*_*ECG12*_, *BsCL*_*ECG12*_, and *ShEn*_*ECG12*_, respectively. A summary of all body surface descriptors of VF progression is reported in Table 2.

**TABLE 2:** Summary of all BSPM markers of VF dynamics used in this study.

**BSPM index**	**Signal property**	**Mathematical definition and references**	**Hypothesis**
*NDI*_*BSPM*_ [%]	Surface f-wave multielectrode spatial complexity	The ratio of the sum of the fourth-to-last SVD eigenvalues to the sum of all eigenvalues (Di Marco et al., 2012; Meo et al., 2018)	More disorganized VF is less accurately described by a 3D dipole (higher *NDI*_*BSPM*_)
*A*_*BSPM*_ [mV]	Surface f-wave amplitude	Median of single-lead f-wave amplitudes, computed as the temporal average of the difference between upper and lower envelopes, obtained by polynomial interpolation of signal local extrema (Meo et al., 2013b)	Lower surface f-wave amplitude reflects more complex VF (lower *A*_*BSPM*_)
*BsCL*_*BSPM*_ [ms]	Surface f-wave cycle length	Median of single-lead f-wave cycle lengths, computed as the mean temporal distance between consecutive local signal maxima (Meo et al., 2013b, 2018)	Higher VF complexity is associated with faster fibrillatory activities on body surface (lower *BsCL*_*BSPM*_)
*ShEn*_*BSPM*_ [a.u.]	Surface f-wave regularity	Expected value of f-wave amplitude self-information (i.e., negative logarithm of f-wave amplitude probability density function) (Chicote et al., 2016; Hajeb-Mohammadalipour et al., 2018)	Less regular and repetitive signal patterns describe more disorganized VF (higher *ShEn*_*BSPM*_)

*The same definitions apply to parameters derived from the equivalent 12-lead ECG (with subscript “BSPM” replaced by “ECG12”); a.u., arbitrary units.*

### Time Analysis of VF Electrical Complexity in Relation to Its Termination Mode and Mechanisms

To explore VF progression over its entire duration in detail and evaluate the ability of signal indices to track intra-episode changes in complexity, each of the presented BSPM- and ECG-metrics has been assessed in consecutive 500-ms frames, so as to obtain only one curve for each index to be straightforwardly compared with the simultaneous time evolution of the multielectrode body surface signals.

On the other hand, to systematically validate the ability of these indices to describe VF complexity in a statistical framework, all body surface parameters have been assessed at specific time frames in all recordings, i.e., at the beginning (“VF start”) and at the end of the VF episode (“VF end”), thus returning one scalar value per VF stage to be then used for subsequent statistical analyses. The selection of the lower bound of the “VF start” window depended on VF initiation mode. Specifically, in case of spontaneous VF, the beginning of the “VF start” frame was taken in correspondence to the onset of the second VF beat. When VF was externally induced, the 4-s signal portion starting at the second VF beat after the last stimulation spike was considered instead. On the contrary, the choice of the “VF end” starting time was related to VF cessation mechanisms, based on the definition of “sustained VF” and “self-terminating VF” from section “Noninvasive Mapping of VF and BSPM Preprocessing.” To examine the dynamics of late VF in the “sustained VF” set, signal features were computed from the “VF end” frame over the last 4 s of the recording with stable isoelectric line before DCC discharge. Conversely, in the “self-terminating VF” group, the terminal “VF end” window covered the last 4 s of VF before rhythm transition. This scheme enabled a rigorous statistical comparison between signal features at different and easily identifiable time points of VF based on the criteria described above, which were the same for all recordings.

As in Meo et al. (2017), each of these episode portions was assumed equal to 4 s, in order to account for a sufficient number of fibrillatory cycles and increase statistical confidence for signal features’ assessment. This choice is also in keeping with previous studies about human VF (Clayton et al., 1995; Haïssaguerre et al., 2018), demonstrating that abrupt changes in VF complexity usually occur within the first 4 to 5 s of arrhythmia, with significant CL acceleration and increased electrogram fragmentation. In the light of this, too short recordings (less than 8 s) were discarded from our analysis. For the same reason, we decided to compare only signal indices from the initial and the terminal 4-s portions of VF, and not to include further intermediate frames between the two of them, as in some recordings it was not possible to extract signal segments with the same duration prior to feature extraction.

At each of the aforementioned VF stages, we investigated whether and how VF termination mode affects body surface complexity. Accordingly, differences between “self-terminating VF” and “sustained VF” were tested at the onset (“VF start”) and at the end (“VF end”) of all episodes. To evaluate the impact of structural alterations on VF electrical patterns, unmatched comparisons were performed between SHD and NSHD patients during early (“VF start”) and late VF (“VF end”). Furthermore, to incorporate the information about VF cessation modality, differences between “self-terminating VF” and “sustained VF” were tested at the onset (“VF start”) and at the end (“VF end”) of the episode in each patients’ population (SHD and NSHD groups).

Finally, to validate the ability of body surface indices to detect temporal changes in VF dynamics, we analyzed a subset of 30 patients, for whom a measure of intracardiac CL was available at the beginning and at the end of VF. In all subjects, an intraepisode acceleration was observed, thus confirming a complexification of the arrhythmia at the level of the tissue. Accordingly, we used this information to investigate whether this transition from early organization (“Organized”) to subsequent disorganization of VF (“Disorganized”) could be captured by the proposed body surface markers, which were therefore computed at both stages of the episode.

### Statistical Analysis

Continuous variables in tables were expressed as mean ± standard deviation, while categorical variables were reported as counting and percentages. Lilliefors test was preliminarily applied to all parameters to verify their distribution. If data followed a Gaussian distributed distribution, intergroup differences were checked by an unpaired Student’s *t*-test with Welch’s correction for unequal group variances and sizes. In the other cases, a Wilcoxon’s rank sum test was performed. Chi-squared test was applied to categorical variables. Specifically, these tests were performed at the onset (“VF start”) and the end (“VF end”) of VF to investigate the link between electrical characteristics and mechanism complexity (i.e., SHD vs. NSHD patients), as well as their relation with VF termination mode (“self-terminating VF” vs. “sustained VF”). Statistical tests were considered significant if *p* < 0.05. Changes in body surface indices between the onset and the offset of VF (“VF start” vs. “VF end”) were assessed by a paired Student’s *t*-test from normally distributed parameters or nonparametric Wilcoxon signed-rank test otherwise. Parameters in the boxplots are displayed as median with interquartile range (IQR).

The same analysis was applied to the subset of patients presented in section “Other Body Surface Markers of VF Electrical Complexity and Dynamics” to validate intraepisode changes in VF dynamics (“Organized” vs. “Disorganized”) from body surface markers as matched with intracardiac CL. Furthermore, transitions from “Organized” to “Disorganized” VF were precisely determined from every signal feature by the receiver operating characteristics curve (ROC) analysis. Specifically, numerical thresholds for these transitions were computed as the cutoff values, which simultaneously maximized the sensitivity (Sens: rate of correct predictions of “Disorganized” VF) and the specificity (Spec: rate of correct predictions of “Organized” VF) of the classifier, thus providing the best discrimination between “Organized” and “Disorganized” stages of VF. This approach allowed us to automatically determine a unique threshold optimized over the entire database and cope with interpatient and intrapatient variability of signal indices. Classifiers’ performance was quantified by the values of sensitivity and specificity associated with the optimal cutoff, as well as the area under the ROC curve (AUC).

## Results

### VF Mapping

Overall, VF was successfully induced in 51 out of 75 episodes in SHD patients (68%), and 130 out of 177 episodes in the NSHD group (74%), with no differences between the two populations (*p* = 0.38). In the remaining cases, it occurred spontaneously, also with similar rates in both patients’ groups. VF was shorter in NSHD (14 ± 8 s) than SHD patients (19 ± 13 s, *p* = 0.003), regardless of its termination mode. Spontaneous cessation of VF prior to shock delivery was observed in 37 out of 177 episodes (21%) in patients with structurally normal hearts, and 13 out of 75 (17%) episodes in the other population, with no significant intergroup differences (*p* = 0.38). Time to self-termination of VF was slightly longer in SHD (14 ± 9 s) than in NSHD patients (9 ± 6 s, *p* = 0.04). On the other hand, VF was terminated by DCC in 60 out of 75 cases (20 ± 13 s) and 140 out of 177 cases (16 ± 8 s) in SHD and NSHD subjects, respectively, also with similar intragroup proportions (*p* = 0.88) and duration (*p* = 0.06). In both groups, sustained VF cases were more numerous than the self-terminating ones (*p* < 0.0001) and required DCC. Furthermore, shocked episodes were longer than those terminating spontaneously both in the SHD (*p* = 0.04) and NSHD (*p* < 0.0001) subjects. At least one episode of VF was mapped in each patient, with comparable proportions between the two groups (SHD 2.3 ± 1.9 vs. NSHD 2.3 ± 2.2 episodes/patient, *p* = 0.52).

### BSPM-Based Analysis of Electrical Complexity Dynamics

An overall increase in VF complexity over time is underlined by all BSPM markers (*p* < 0.0001). At early stage, organized VF is measured by low NDI (VF start 1.9 ± 1.4% vs. VF end 3.4 ± 2.4%), amplitude voltage *A*_*BSPM*_ is high (0.5 ± 0.3 vs. 0.3 ± 0.2 mV), and slow activities are measured by long *BsCL*_*BSPM*_ (166 ± 21 vs. 154 ± 22 ms) in the entire dataset. An unexpected, albeit significant, trend of *ShEn*_*BSPM*_ is observed in the entire dataset, with a decrease in time from 6.6 ± 0.5 to 6.2 ± 0.6, in contrast with our initial hypothesis.

**FIGURE 1:**
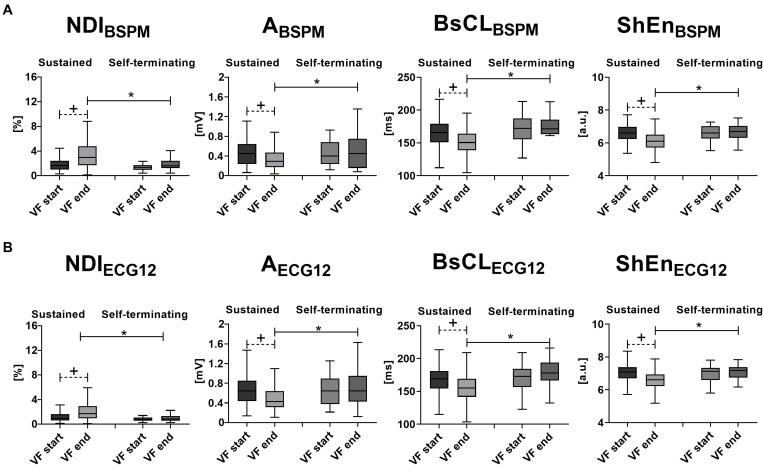
Body surface signal features’ time evolution and VF termination: statistical analysis of intraepisode changes (“VF start” vs. “VF end,” matched *p* value) in VF organization for a specific VF termination mode, and evaluation of differences between “Sustained VF” and “Self-terminating VF” at each episode phase (unmatched *p-*value) using body surface signal features. **(A)** BSPM-derived metrics of VF dynamics (from left to right): *NDI*_*BSPM*_, *A*_*BSPM*_, *BsCL*_*BSPM*_, and *ShEn*_*BSPM*_. **(B)** ECG-derived metrics of VF dynamics (from left to right): *NDI*_*ECG12*_, *A*_*ECG12*_, *BsCL*_*ECG12*_, and *ShEn*_*ECG12*_. Parameters in the boxplots are displayed as median with IQR. ^+^*p* < 0.05 vs. VF start (matched comparison, dashed line); *p < 0.05 vs. sustained VF (unmatched comparisons, continuous line); a.u., arbitrary units.

As shown in Figure 1A, at the onset of VF, none of these indices can significantly distinguish between sustained and self-terminating VF, while at the end of DCC-shocked episodes complexity is significantly higher, and amplitude and entropy appear reduced, with faster oscillations than in case of spontaneous termination (*p* < 0.0001). Furthermore, time evolution of sustained VF is characterized by more complex and variable waveforms, which are significantly captured by all BSPM markers. By contrast, self-terminating VF results in more regular and simple patterns and a stable trend in signal features. After removing outliers identified by the Grubb’s test (with significance taken for *p* < 0.05), a modest increasing correlation between the length to self-termination from VF onset and *NDI*_*BSPM*_ fold change from “VF start” to “VF end” is observed in our database (Pearson’ correlation coefficient *R* = 0.56, *p* = 0.003), while the other BSPM features do not exhibit any significant correlation.

Temporal increase in body surface complexity is also corroborated by a decrease in intracardiac CL (Organized 197 ± 24 ms vs. Disorganized 169 ± 20 ms; *p* < 0.0001) in the validation subset introduced in section “Other Body Surface Markers of VF Electrical Complexity and Dynamics,” as confirmed by the outcome of the paired analysis and ROC assessment in Table 3 (*p* < 0.0001 for all BSPM parameters, except for *A*_*BSPM*_, *p* = 0.0003).

**TABLE 3:** Statistical analysis of intraepisode changes in VF organization from body surface signal analysis of VF episodes with simultaneous invasive measure of VF CL.

	**Organized**	**Disorganized**	***p*-value**	**AUC**	**Sens**	**Spec**
*NDI*_*BSPM*_ [%]	1.20.7	2.51.9	**<0.0001**	78	90	57
*NDI*_*ECG12*_ [%]	0.80.5	1.81.3	**<0.0001**	79	77	70
*A*_*BSPM*_ [mV]	0.60.2	0.30.2	**0.0003**	70	77	60
*A*_*ECG12*_ [mV]	0.80.3	0.60.3	**0.001**	71	67	77
*BsCL*_*BSPM*_ [ms]	17920	16216	**<0.0001**	77	53	93
*BsCL*_*ECG12*_ [ms]	17816	16018	**<0.0001**	79	73	80
*ShEn*_*BSPM*_ [a.u.]	6.90.4	6.30.5	**<0.0001**	79	80	67
*ShEn*_*ECG12*_ [a.u.]	7.10.4	6.70.5	**<0.0001**	76	70	77

*All BSPM- and ECG-derived metrics are reported as mean ± standard deviation; *p*-values in boldface are statistically significant vs. “organized.” AUC, area under curve; Sens, sensitivity; Spec, specificity; a.u., arbitrary units.*

Late disorganization of VF is accurately assessed according to the statistical tests and ROC analysis of signal indices, although the ratio between sensitivity and specificity is slightly unbalanced for *NDI*_*BSPM*_ and *ShEn*_*BSPM*_. Also, *BsCL*_*BSPM*_ sensitivity is relatively poor than for other parameters.

**FIGURE 2:**
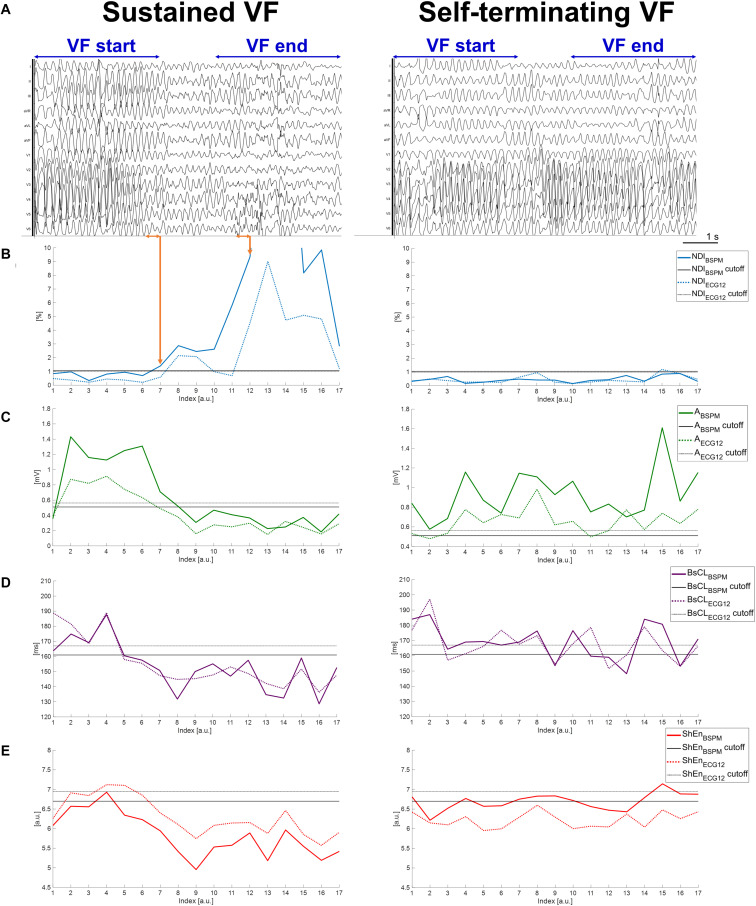
Temporal dynamics of sustained and self-terminating VF and body surface metrics of complexity. **(A)** A representative equivalent 12-lead ECG computed from measured BSPMs during an episode of sustained VF (left side of the panel) and during self-terminating VF (right side). Only signals recorded during VF episodes matching the criteria described in section “Noninvasive Mapping of VF and BSPM Preprocessing” and “Time Analysis of VF Electrical Complexity in Relation to Its Termination Mode and Mechanisms” were exported from the mapping system for subsequent quantitative analysis, i.e., those in the time frame between the first time sample of the “VF start” window (i.e., at the onset of the second VF beat of spontaneous or induced events), and the last time sample of the “VF end” window (i.e., before DCC shock for sustained VF, or before transition to sinus rhythm for self-terminating VF). The “VF start” and “VF end” time frames used for signal feature statistical analysis are indicated on the top of the ECGs for each episode. Each curve value is associated with VF patterns from the previous 500-ms frame on surface signals over the entire duration of the episode. The most abrupt pattern changes during sustained VF are indicated by orange arrows. **(B)** Time evolution of *NDI*_*BSPM*_ and *NDI*_*ECG12*_. **(C)** Time evolution of *A*_*BSPM*_ and *A*_*ECG12*_. **(D)** Time evolution of *BsCL*_*BSPM*_ and *BsCL*_*ECG12*_. **(E)** Time evolution of *ShEn*_*ECG12*_ and *ShEn*_*ECG12*_. Continuous and dotted line curves are associated with signal features derived from BSPMs and equivalent ECGs, respectively. Colored lines represent the temporal trend of parameter, with each point describing VF dynamics in the previous 500-ms signal frame. Horizontal continuous (dotted) black lines are the thresholds output by ROC analysis of each BSPM (ECG) index, validated by comparison with the intracardiac CL.

To gain deeper insights into the ability of these indices to describe VF dynamics, equivalent ECGs extracted from BSPMs from a representative example for each VF termination mode are shown in Figure 2, as well as the time evolution of all body surface markers and the thresholds returned by the ROC analysis.

Concerning the sustained VF episode in Figure 2A (left), from the NDI index even short transients of organization/disorganization can be detected using optimized thresholds (Figure 2B). The timing of irreversible disorganization before cardioversion (approximately 6 s from the onset) can be can promptly identified from body surface (in less than 3 s), with more pronounced differences between the beginning and the end of the episode underlined by the BSPM-derived index (a difference of one order of NDI magnitude) rather than the 12-lead counterpart. On the other hand, in case of spontaneous termination of VF as in Figure 2A (right), the range of values covered by the NDI parameter over time is less broad (Figure 2B, right), and always below the ROC thresholds determined from the validation set. Similar dynamics and timings can be retrieved by descriptors of f-wave amplitude in Figure 2C (left) for the shocked episode, and again more marked changes from the onset can be better appreciated from BSPMs rather than ECG. In contrast, both *A*_*BSPM*_ and *A*_*ECG12*_ parameters are characterized by a stable evolution when VF terminates with no external intervention Figure 2C (right). Similar considerations can be done for body surface CL in Figure 2D regardless of the termination mode, although the trend of indices computed using different lead configurations are slightly different at certain time frames. These differences are more evident during DCC-terminated (Figure 2D, left) rather than nonsustained VF (right). Unlike the other parameters, the timing of disorganization of shocked VF in Figure 2E (left) seems to be better explained by Shannon entropy from ECG rather than BSPM. In contrast, during self-terminating VF (Figure 2E, right), *ShEn*_*BSPM*_ curve crosses the ROC cutoff several times, and *ShEn*_*ECG12*_ is always below its corresponding threshold, i.e., indicating spurious increased complexity.

### Relation Between Electrical Complexity of VF From BSPMs and Structural Alterations of the Myocardium

**FIGURE 3:**
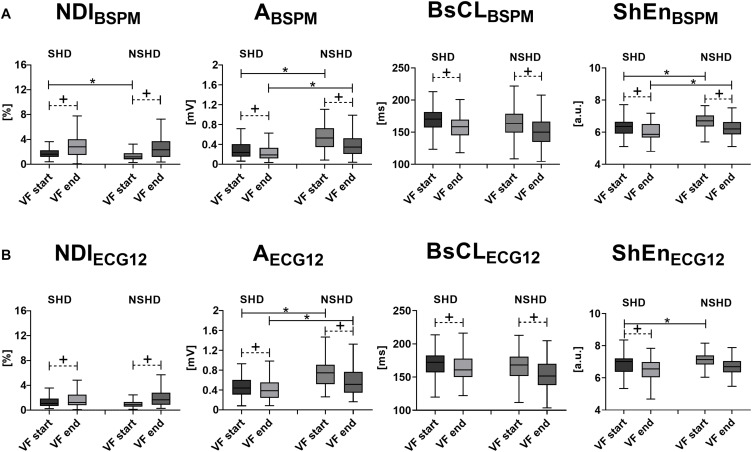
Body surface electrical complexity and structural abnormalities during VF. Statistical analysis of intraepisode changes (“VF start” vs. “VF end,” matched *p-*value) in VF organization in SHD and NSHD patients and assessment of intergroup differences at the starting and the offset of the episode (unmatched *p-*value) using body surface signal features. **(A)** BSPM-derived metrics of VF dynamics (from left to right): *NDI*_*BSPM*_, *A*_*BSPM*_, *BsCL*_*BSPM*_ and *ShEn*_*BSPM*_. **(B)** ECG-derived metrics of VF dynamics (from left to right): *NDI*_*ECG12*_, *A*_*ECG12*_, *BsCL*_*ECG12*_ and *ShEn*_*ECG12*_. Parameters in the boxplots are displayed as median with IQR. ^+^*p* < 0.05 vs. VF start (matched comparison, dashed line); *p < 0.05 vs. sustained VF (unmatched comparisons, continuous line); a.u., arbitrary units.

As underlined by the statistical analysis of BSPM metrics in Figure 3A, according to NDI, in SHD patients VF is initially more complex than in NSHD patients (*p* = 0.0007). Similarly, f-wave amplitude is slightly lower in patients with structural alterations (*p* < 0.0001), with moderately slower cycles (*p* = 0.06). Entropy is also lower at early stage of VF in these patients (*p* < 0.0001). In both populations, there is a strong increase in electrical complexity with progression of VF (*p* < 0.0001). At the end of the episode, marked intergroup differences in amplitude and entropy are still preserved (*p* < 0.0001). In contrast, body surface CL (*p* = 0.06) and NDI (*p* = 0.54) are comparable at the end of VF in both datasets.

**FIGURE 4:**
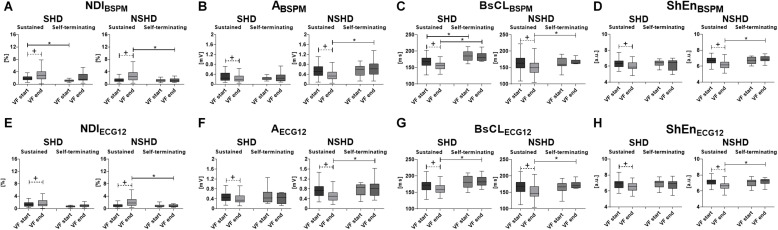
Body surface electrical complexity in relation with mode of termination of VF and structural abnormalities. Statistical analysis of intraepisode changes (“VF start” vs. “VF end,” matched *p-*value) in VF dynamics in SHD (left side of each panel) and NSHD (right side) patients and differences between sustained and self-terminating VF at the starting and the offset of the episode (unmatched *p*-value) using BSPM- and ECG-derived parameters: **(A)** *NDI*_*BSPM*_; **(B)** *A*_*BSPM*_; **(C)** *BsCL*_*BSPM*_; **(D)** *ShEn*_*BSPM*_; **(E)** *NDI*_*ECG12*_; **(F)** *A*_*ECG12*_; **(G)** *BsCL*_*ECG12*_; **(H)** *ShEn*_*ECG12*_. Parameters in the boxplots are displayed as median with IQR. ^+^*p* < 0.05 vs. VF start (matched comparison, dashed line); *p < 0.05 vs. sustained VF (unmatched comparisons, continuous line); a.u., arbitrary units.

In the presence of structural abnormalities, lower complexity at baseline is assessed by lower NDI (*p* = 0.004, Figure 4A, left) and slower surface CL (*p* = 0.008, Figure 4C, left) from BSPMs, which are associated with spontaneous cessation of VF and have a stable evolution during the episode. In contrast, amplitude (Figure 4B, left) and entropy-based (Figure 4D, left) indices do not exhibit any significant correlation with VF self-termination (*p* = 0.98 and *p* = 0.82, respectively). On the other hand, sustained VF is characterized by a significant increase in electrical disorganization and outlined by all BSPM markers. At the end of the episode, all indices are comparable, regardless of the termination mode, but cycles are still faster in patients undergoing DCC (*p* = 0.002).

In patients with structurally intact hearts (NSHD, Figures 4A–D, right side of the panels), self-termination of VF cannot be assessed by BSPM markers at the onset of VF, as complexity is comparable in shocked and self-terminating episodes. However, unlike the SHD group, during sustained VF a sharper increase in electrical complexity (Figure 4A, right), with progressive decrease in f-wave amplitude (Figure 4B, right), surface CL (Figure 4C, right) and entropy (Figure 4D, right), is underlined by all parameters (*p* < 0.0001). Furthermore, VF is more disorganized at the end of episodes requiring DCC than those terminating with no external intervention (*NDI*_*BSPM*_, *p* = 0.0095), with low-voltage (*A*_*BSPM*_, *p* = 0.002), fast oscillations (*BsCL*_*BSPM*_ *p* = 0.004).

### Comparison With 12-Lead ECG-Derived Metrics

As previously explained, the BSPM descriptors of VF dynamics introduced in section “Materials and Methods” have also been computed from an equivalent standard ECG to verify whether it is possible to derive similar information about VF progression from a reduced number of electrodes.

As for BSPM parameters, all ECG-derived indices highlight increased VF complexity, a decline in f-wave amplitude, and CL shortening between the beginning (VF start, *NDI*_*ECG12*_: 1.3 ± 1.0%; *A*_*ECG12*_: 0.7 ± 0.3 mV; *BsCL*_*ECG12*_: 168 ± 22 ms; *ShEn*_*ECG12*_: 7.0 ± 0.5) and the end of the episode (VF end, *NDI*_*ECG12*_: 1.9 ± 1.5%; *A*_*ECG12*_: 0.5 ± 0.3 mV*BsCL*_*ECG12*_: 158 ± 23 ms; *ShEn*_*ECG12*_: 6.6 ± 0.6, *p* < 0.0001).

Similarly, at the beginning of VF, there are no significant differences between sustained and self-terminating episodes (Figures 1A,B). In keeping with BSPM analysis, sustained VF is associated with an intraepisode increase in complexity *NDI*_*ECG12*_, a reduction in f-wave amplitude *A*_*ECG12*_ and entropy *ShEn*_*ECG12*_, and a CL shortening according to *BsCL*_*ECG12*_ (*p* < 0.0001). On the other hand, ECG parameters do not change significantly between the beginning and the end of self-terminating VF. Again, we find a moderate correlation between *NDI*_*ECG12*_ fold change and length to episode self-termination (Pearson’s correlation coefficient *R* = 0.44, *p* = 0.03), as opposed to other ECG features. In addition, unlike cases of spontaneous termination, late sustained VF is more disorganized, as outlined by higher *NDI*_*ECG12*_ (*p* = 0.0003) and lower *ShEn*_*ECG12*_ (*p* < 0.0001), with faster fibrillatory patterns (*BsCL*_*ECG12*_, *p* < 0.0001) and smaller wave deflections (*A*_*ECG12*_, *p* = 0.009).

Changes in VF dynamics are correctly captured by ECG metrics and validated by simultaneous measure of intracardiac CL (*p* < 0.0001 for all ECG indices, except for *A*_*ECG12*_, *p* = 0.001). As shown in Table 3, organized and disorganized stages of VF can be accurately identified, and prediction performance still appears satisfactory when a reduced number of leads is considered. A more careful inspection of the temporal evolution of each ECG feature in Figure 2 reveals that ECG-derived metrics overall follow quite well their BSPM counterparts, and they can identify the timing of VF disorganization during shocked episodes, while showing a stable evolution in case of self-termination. However, differences between the onset and the end of VF according to ECG indices are usually less pronounced that those highlighted from BSPMs. Once again, *NDI*_*ECG12*_ and *A*_*ECG12*_ can capture small changes in VF temporal patterns and their trends are similar to those computed from BSPMs. The CL index *BsCL*_*ECG12*_ often exhibits a different evolution compared with *BsCL*_*BSPM*_, but disorganization timing can be still captured within the first seconds of VF. On the contrary, the resolution of *ShEn*_*ECG12*_ is overall lower than the other indices and ROC thresholds are not optimal in all patients.

In contrast with BSPM analysis, at the onset of VF differences between populations with different structural characteristics are not captured by all parameters (Figure 3B). Specifically, *NDI*_*ECG12*_ is comparable in both sets at baseline (*p* = 0.07), and fibrillatory activities have similar cycles (*p* = 0.13). In contrast, in patients with normal hearts higher initial organization is rendered by lower entropy (*p* = 0.002), and f-wave amplitude is higher (*p* < 0.0001) than in SHD subjects. Both groups exhibit a significant intraepisode increase in complexity, a reduction in f-wave amplitude, and surface CL shortening, although more modest in patients with structural abnormalities and compared with BSPM-derived trends. When VF terminates, ECG markers enhance similar behaviors to BSPM indices, i.e., low voltage oscillations (*p* < 0.0001), but slow cycles in SHD patients (*p* = 0.002). On the contrary, both *NDI*_*ECG12*_ and *ShEn*_*ECG12*_ do not account for the effect of structural alterations at the end of VF (*p* = 0.27 and *p* = 0.09, respectively).

The relation between VF termination mode and structural alterations has also been investigated from ECG, and the results of this analysis are reported in Figures 4E–H. Unlike their BSPM counterpart, ECG-based indices cannot significantly predict self-termination of VF in the SHD population, as there were no differences between nonsustained and sustained VF events at baseline. In SHD patients, episodes of sustained VF are still associated with an increase in electrical complexity (Figure 4E, left) and decline in amplitude of the fibrillatory patterns with time (Figure 4F, left), although with a more blunted dynamics (*NDI*_*ECG12*_ and *A*_*ECG12*_, *p* = 0.005). ECG feature trend is again stable in case of spontaneous termination, with no significant changes in all parameters between the onset and the end of VF in the SHD group. Differences between sustained and self-terminating episodes in terms of surface CL (Figure 4F, left, *p* = 0.0008) are preserved during late VF. In the NSHD group, ECG markers of VF organization highlight similar patterns to those obtained by BSPM analysis. According to all ECG parameters, differences in body surface complexity between sustained and non-sustained episodes clearly appear only later in time (“VF end”), whereas at the onset of VF (“VF start”) ECG features are comparable in both datasets. As for BSPM indices, a strong reduction in electrical organization (i.e., with an increase in NDI, Figure 4E, right), f-wave amplitude (Figure 4F, right), CL (Figure 4G, right) and entropy (Figure 4G, right) can be observed during sustained VF exclusively (*p* < 0.0001).

## Discussion

This study put forward a noninvasive methodology to explore and measure the complexity of VF by analyzing body surface cardiac potentials at multiple locations and finer spatial resolution. To our knowledge, this is the first report that explicitly and noninvasively investigates the link between patterns of electrical complexity of VF and myocardial structural alterations in a large cohort of patients using a multilead signal processing approach.

### Temporal Evolution of Electrocardiographic Markers of VF Organization

As in Tovar and Jones (2000), Haïssaguerre et al. (2018), and Robson et al. (2018), overall we observed a sharp transition from early organization to late disorganization of electrical patterns of VF, due to the progressive and rapid metabolic deterioration of myocardial tissue, which drastically reduces the probability of survival to cardiac arrest and defibrillation success (Brown et al., 1989; Winkle et al., 1990). In our database, these changes could be captured from body surface signal analysis within a few seconds after the onset of VF (usually less than four), and they were validated with simultaneous measure of endocardial CL in a subset of patients. Furthermore, the dynamics of VF could be tracked in high detail, and the contribution of signals from multiple electrodes could be effectively summarized into one curve per parameter, thus easing the inspection of the fibrillatory patterns.

When information about VF termination mode was included as a fixed effect into the statistical analysis, distinct dynamic behaviors were underlined by body surface signal processing. All electrocardiographic parameters described stable fibrillatory patterns during self-terminating VF, as confirmed by previous invasive studies (Mäkikallio et al., 2002; Cismaru et al., 2013), whereas a strong increase in electrical complexity characterized episodes requiring DCC, despite comparable degrees of complexity at baseline.

Temporal increase in NDI assessed by SVD significantly correlated with progressive disorganization of VF with time. Several studies have confirmed the prognostic value of SVD-based analysis of body surface cardiac electrical activity. SVD of T-wave from surface ECG has been successfully applied to assess physiological and pathological properties of ventricular repolarization (Priori et al., 1997; Malik et al., 2000), under the hypothesis that increased dispersion of the electrical recovery would have been less accurately modeled by a 3D dipole. The study presented in Meo et al. (2018) moved from similar assumptions to measure the complexity of atrial fibrillation and use this information to guide ablation therapy. Our findings confirm the applicability of this methodology to cardiac signals during VF, and activation patterns can be reliably quantified by NDI from body surface.

In our VF population, f-wave amplitude decreased with progression of VF, in line with (Caldwell et al., 2012). As surface ECG can be regarded as the sum of all of the underlying myocardial electrical fields, the level of organization of the VF waveform relates to the organization of the myocardial elements in relation to each other. Local electrical vectors will increasingly oppose each other, thereby reducing the electrical voltage as measured at a global level (Wltkowskl et al., 1998; Balderston et al., 2018). Nevertheless, amplitude-related measures of VF complexity should be examined with caution, as their values can be affected by other factors not related to the arrhythmia itself, such as body size and cardiac axis (Ng and Goldberger, 2014). Furthermore, not only pairwise comparisons between intraepisode amplitude measures may be hampered by sudden changes in contact quality of the recording electrodes, but also the intrinsic variability of amplitude values from patient to patient may affect the reliability of this metric alone with respect to the interpretation of VF dynamics.

As in Cismaru et al. (2013), surface VFCL prolonged between the onset and the end of self-terminating VF, while it decreased during sustained VF. VFCL from body surface potentials can provide an assessment of temporal changes in the properties of cellular substrate during VF (Patwardhan et al., 2000), and it is used as a predictor of defibrillation outcome (Strohmenger et al., 2001). Unlike previous works, surface CL was computed through a peak detection-based algorithm rather than a frequency domain approach (e.g., dominant frequency from Fourier transform), as stationarity is a fundamental assumption of the Fourier transform, thus spectral analysis may fail to capture the dynamical nature of VF (Cheng et al., 2012).

We expected entropy to increase with VF progression, as in Hajeb-Mohammadalipour et al. (2018). Yet, in some studies low values of several entropy-based metrics have also been measured even from ECG rhythms requiring external DCC (Chicote et al., 2016; Oh et al., 2017), and, more generally, during specific pathological conditions, such as congestive heart failure (Acharya et al., 2017). This apparent contradiction can be potentially explained by two reasons: (1) some entropy indices (e.g., Shannon entropy) are signal amplitude-dependent, thus even in presence of extremely regular patterns (such as sinus rhythm) they may not correctly capture signal regularity, if voltage is too low (Oh et al., 2017); (2) prior to entropy assessment, ECG signals can be preprocessed using different approaches [e.g., wavelet transform (Acharya et al., 2015; Oh et al., 2017)] in order to enhance specific patterns, therefore in some contexts results may differ from those obtained from raw data and make a direct comparison more difficult. Because in our implementation the entropy index suffered from poor temporal resolution, and it was often unable to track subtle intraepisode changes in VF progression, special attention should be paid to the interpretation of quantitative results in our database.

### Electrical Complexity and Structural Cardiac Diseases

VF mechanisms are still poorly understood, thus hampering the development of suitable therapeutic strategies and arrhythmia management. Despite the apparent lack of synchrony and coordination of the fibrillatory process, several studies have confirmed that there is indeed a high degree of temporal and spatial organization in the fibrillating tissue (Gray et al., 1998). Nonetheless, we still lack tools to characterize VF electrical activation patterns and capture subtle changes both in time and space across the myocardium. Our research demonstrates that organization of VF can be measured from body surface, and that it accounts not only for the electrical properties of the myocardium, but also for those of the structural substrate.

SHD patients globally exhibited higher complexity than those with normal hearts at early stage of VF. Specifically, in this group lower f-wave amplitude and slower surface CL have been measured from body surface potentials, which is in line with previous research on ischemic VF, although the leading mechanisms have not been clearly elucidated yet (Bradley et al., 2011; Caldwell et al., 2012). The amount of nondipolar components as quantified by NDI was also initially higher in the SHD population, suggesting that structural substrate also contributes to electrical organization and cannot be accurately modeled in a 3D space. Although VF was initially more organized in the NSHD group, in these patients the temporal increase in VF complexity was more pronounced, rapid, and abrupt than in those with structural alterations, whereas in the SHD group structural substrate seems to have a dampening effect on VF electrical perpetuation and make it evolve more gradually. Indeed, during late VF the influence of structure appears less relevant, as BSPM complexity was comparable in both groups. The modalities according to which electrical-related factors may overdrive structural alterations with progression of VF are not clear though, and deeper insights may be provided by phase mapping (Umapathy et al., 2010; Robson et al., 2018).

Importantly, increased baseline complexity of VF as resulting from the combination of structural and non-structural alterations was found to be associated with sustained VF episodes from SHD patients, and it prompted DCC performance to terminate the arrhythmia. More precisely, slow fibrillatory activities at baseline predicted spontaneous termination of VF, as confirmed invasively by Muñoz et al. (2009). Increased electrical disorganization at the beginning of VF was also quantified by a higher amount of nondipolar content (i.e., higher NDI), which is known to be associated with significant arrhythmic complications in a multitude of pathological conditions (Priori et al., 1997; Malik et al., 2000). However, to our knowledge, our research is the first application of NDI as a noninvasive tool to predict VF termination mode in the first seconds of the arrhythmia, thus opening meaningful perspectives into the understanding and management of VF. Similarly, the descriptive accuracy of f-wave amplitude and entropy has not been systematically assessed yet in this framework, and our report demonstrates that both parameters are poor descriptors of VF self-termination at baseline in all SHD patients. The role of amplitude-based metrics from surface ECG in the characterization of ischemic VF has been questioned (Caldwell et al., 2012), therefore any evidence provided by these parameters should be investigated in more detail.

In patients with structurally normal hearts, VF had a similar evolution to the SHD group depending on its termination mode, although intraepisode changes in body surface complexity were characterized by a faster and sharper evolution than in the presence of structural diseases. All electrocardiographic features exhibited similar trends, in keeping with previous research (Cismaru et al., 2013). However, it is worth to note that differences between sustained and self-terminating episodes according to all BSPM parameters arose only during late VF (i.e., approximately 10 s after arrhythmia onset in the NSHD group), hinting at potential additional mechanisms driving VF dynamics beyond structural substrates in this population. Also in this case, a correlation of BSPM-derived indices with the distribution of VF drivers (Haïssaguerre et al., 2018) would be desirable to better characterize the electrophysiological causes of VF in these patients.

### What Can Be Inferred From Standard ECG?

As mentioned in section “Other Body Surface Markers of VF Electrical Complexity and Dynamics,” all BSPM features have also been computed from an equivalent standard ECG to investigate the scalability of our findings to the 12-lead configuration, which is more easily available in clinical centers. Overall, our results show that the characterization of VF dynamics is still preserved by the alternative signal features, although transitions in electrical organization appear less exacerbated by ECG indices during sustained VF compared to their BSPM counterparts. The reduced spatial resolution had a significant impact on features’ computation in SHD patients, for whom the ability of certain indices (i.e., surface CL and NDI) to predict self-termination of VF at baseline was lost. This evidence confirms the benefits from a multilead signal processing approach, as the analysis of multiple electrodes may help: (1) increasing the statistical confidence of body surface measures; (2) compensating the lack of information from standard leads in case of electrode displacement, poor signal quality, etc., which would be otherwise impossible to derive and (3) capturing cardiac electrical activity at multiple angles and orientations that are not generally accounted for by standard ECG configurations. With regard to that, the use of posterior leads has proven to be useful in the localization of premature contractions from the outflow tracts in Zhang et al. (2017), and nonstandard systems (e.g., the 15-lead ECG) should be generally considered in patients who also present inferior and suspected posterior myocardial infarction (Steg et al., 2012). Finally, multielectrode potential mapping may be simultaneously used with other techniques (for instance, electrocardiographic imaging) to identify diseased ventricular sites, which may induce threatening arrhythmias, thus combining BSPM timing information with trigger spatial location. This would provide added benefit for patients with apparently normal hearts, but with microstructural alterations that are often misdetected by current imaging techniques (Haïssaguerre et al., 2018).

### Limitations and Perspectives

The limited number of noninvasive signal processing approaches to measure VF complexity from literature made the comparison between BSPM parameters more challenging. The studies mentioned in our report have been performed in a large variety of experimental frameworks and using different models, thus evidence from the state of the art appeared sometimes controversial. However, our findings are in line with previous research about human VF, and they have been corroborated by invasive measures, thus increasing the confidence of our conclusions.

To validate surface signal assessment, we used endocardial CL as a reference, since it is conventionally regarded in clinics as a good marker of electrical activation rate at the level of the tissue. However, for ethical reasons, it was not possible to explore the mechanisms of VF maintenance at a higher resolution in this clinical scenario. In Meo et al. (2020), we have tried (at least partially) to address this question, and we have shown that in explanted porcine hearts NDI can identify more heterogeneous repolarization substrates, with uneven spatial distribution of epicardial CL and reentrant drivers from ventricular sites with different repolarization timings. Further investigation is needed to translate these findings from our experimental model to clinical patients, and to test additional hypotheses to explain all the pathologies encountered across our dataset.

No specific gender-related differences in VF dynamics were found in our dataset based on surface signal analysis. Overall, we observed that VF progression was comparable both in male and female patients according to all parameters, independently of the lead configuration. Further investigation is needed to elucidate the mechanisms underlying potential inter-gender differences in subpopulations from our dataset and link them to other risk factors.

Our patients’ cohort has been split into two macro-categories (i.e., SHD vs. NSHD), although each group includes patients with different pathologies and electrocardiographic manifestations. This choice is motivated not only by the will to investigate the impact of structural alterations in a more general framework, but also by the reduced number of VF recordings in certain categories (even one or two for certain diseases), which hampers the generalization of certain findings. The inclusion of additional signal recordings in those categories in a future study may allow for the assessment of more specific patient-related electrical signatures during VF.

Body surface markers of VF complexity were not correlated with defibrillation shock outcome, as it goes beyond the scope of this research. Indeed, while the use of hundreds of electrodes is not considered feasible when prompt defibrillation is required for out-of-hospital cardiac arrest, our analysis of VF in a controlled environment proved the descriptive value of multilead electrocardiographic markers and the possibility to extract valuable information about VF complexity using electrical signals only, with very short duration and no need for other information about the patient (e.g., clinical history, anatomy, etc.). In future, we may potentially try to incorporate other noninvasive markers (e.g., ventricular volumes from echocardiography, scar and fibrosis distribution from magnetic resonance imaging, etc.) to verify whether they can provide any additional information beyond electrical markers.

Importantly, some of our findings could be retrieved from standard ECG as well, thus opening promising perspectives to the analysis of VF from body surface in a larger clinical scenario. Nonetheless, as discussed in section “What Can Be Inferred from Standard ECG?”, some important differences between BSPM and ECG features were observed, in particular at the onset of VF, i.e., the impossibility to predict self-termination of VF in patients with structural anomalies from the standard leads, and using a higher number of electrodes may also offer additional insights in the spatial localization of triggering mechanisms. Therefore, future research may try to compensate them, e.g., by introducing novel signal features, or designing alternative ECG lead placement configurations, and/or with a different number of electrodes at specific locations. With regard to that, alternative multilead setups with an intermediate number of electrodes between BSPM and ECG could not be straightforwardly tested. Indeed, for some of the reasons presented in section “Noninvasive Mapping of VF and BSPM Preprocessing”, in certain patients cardiac potentials could not be recorded over the entire set of electrodes (28% of VF episodes, with between 236 and 251 electrodes used). Most of these electrodes were localized in the lower section of the vest, thus comparison with ECG was still feasible in all patients, but a comparison of signal features from nonstandard electrodes was not implementable in a unified framework. For this purpose, in future the use of computational models may help evaluating the robustness of signal indices to different electrode locations and easily varying the number of recording channels while keeping comparable and controlled testing conditions.

Our investigation merely aimed to globally assess progression of VF from body surface, but driving mechanisms of the arrhythmia and their spatial distribution over the ventricles have not been described. As pointed out in section “Discussion,” correlating BSPM metrics with phase mapping analysis may help addressing some unanswered questions and may be attempted in future. At this stage, we showed that body surface mapping can precociously help identifying patients with abnormal electrical disorganization at the onset of VF, who can be therefore subsequently inspected in more detail through more invasive approaches.

## Conclusion

We developed a noninvasive multilead signal processing framework to measure VF complexity, track its temporal evolution and assess the relation between myocardial electrical characteristics and structural substrate. The main findings outlined by our investigation can be summarized as follows:

a. Sustained VF is characterized by an early, sharp increase in electrical complexity, with quick deterioration within the first few seconds from the onset. Conversely, self-terminating VF exhibits a stable evolution throughout the duration of the episode, which can be timeously predicted as well. These different dynamic behaviors hint at distinct mechanisms underlying the perpetuation of the arrhythmia with time.
b. While the termination mode similarly affects VF evolution in all patients regardless of the structural substrate properties, in patients with apparently normal hearts VF is more organized at baseline, but electrophysiological degeneration into sustained forms is more rapid and severe than in those with structural diseases, as myocardial alterations contribute to dampen and slow down VF dynamics. These elements suggest that while structural determinants for VF evolution play a more relevant role at early stage of the arrhythmia, late VF is dominated by alternative driving mechanisms accelerating its deterioration.
c. The combination of structural and electrophysiological substrate characteristics influences the timing of the transition from early organization to late disorganization of VF, and this information may help optimizing defibrillation shock timing and waveforms, thus improving current strategies for VF treatment and opening a perspective toward personalized therapy of this arrhythmia.
d. These distinct patterns of VF complexity can be described in detail by our approach using electrical signals only, and the spatial content of multielectrode BSPMs can be condensed into a few parameters, thus easing visualization and interpretation of VF dynamics from surface cardiac potentials.
e. Some of these results can be retrieved by our approach from the 12-lead ECG as well, thus broadening its applicability to a wider clinical scenario, when high-resolution BSPM systems are not available. Nevertheless, particular attention should be paid to patients without apparent structural hearts diseases, as in these subjects the ECG-based assessment of VF complexity from a lower number of electrodes may be less accurate at early stage of VF compared to the full BSPM configuration and lead to potential misinterpretation of the arrhythmic patterns.

Overall, our signal processing methodology provides relevant insights into the understanding and the management of VF patients.

## Data Availability Statement

Due to the nature of this research, participants of this study did not agree for their data to be shared publicly, so the raw data supporting the conclusions of this article is not publicly available. Requests to access the datasets should be directed to MiH, michel.haissaguerre@chu-bordeaux.fr.

## Ethics Statement

The studies involving human participants were reviewed and approved by Comité de Protection des Personnes Sud-Ouest et Outre Mer III and Institutional Clinical Research and Ethics Committee. The patients/participants provided their written informed consent to participate in this study.

## Author Contributions

All authors have significantly contributed to this work. MM conceived and designed the study, implemented the signal processing methods, analyzed and interpreted the results, and drafted the manuscript. AD, FS, JD, and GC performed VF ablation procedures and helped analyzing clinical data. SP acquired clinical BSPM recordings. PJ, MéH, and MiH supervised clinical data acquisition and helped assessing algorithm performance. LB and OB provided further contributions to the interpretation of the results. RD contributed to the conception of the study, provided feedback about the implementation of the methods and the interpretation of the results, and revised the manuscript.

## Conflict of Interest

The authors declare that the research was conducted in the absence of any commercial or financial relationships that could be construed as a potential conflict of interest.

## Abbreviations

BSPM: body surface potential mapping
CL: cycle length
DCC: electrical cardioversion
DCM: dilated cardiomyopathy
ECG: electrocardiogram
ERP: early repolarization pattern
ERS: early repolarization syndrome
HCM: hypertrophic cardiomyopathy
IQR: interquartile range
LVD: left ventricular dysfunction
NDI: Nondipolar Component Index
NSHD: no structural heart disease(s)
OHCM: obstructive hypertrophic cardiomyopathy
ROC: receiver operating characteristic
SCD: sudden cardiac death
SHDs: structural heart disease(s)
SVD: singular value decomposition
VF: ventricular fibrillation
VT: ventricular tachycardia.

## References

[B1] Acharya U. R. Fujita H. Sudarshan V. K. Oh S. L. Adam M. Tan J. H. (2017). Automated characterization of coronary artery disease, myocardial infarction, and congestive heart failure using contourlet and shearlet transforms of electrocardiogram signal. *Knowl. Based Syst.* 132 156–166. 10.1016/j.knosys.2017.06.026

[B2] Acharya U. R. Fujita H. Sudarshan V. K. Sree V. S. Eugene L. W. J. Ghista D. N. (2015). An integrated index for detection of Sudden Cardiac Death using discrete wavelet transform and nonlinear features. *Knowl. Based Syst.* 83 149–158. 10.1016/j.knosys.2015.03.015

[B3] Al-Khatib S. M. Stevenson W. G. Ackerman M. J. Bryant W. J. Callans D. J. Curtis A. B. (2017). AHA/ACC/HRS guideline for management of patients with ventricular arrhythmias and the prevention of sudden cardiac death: executive summary. *Circulation* 138 e210–e271. 10.1161/CIR.0000000000000549 29084733

[B4] Balderston J. R. Gertz Z. M. Ellenbogen K. A. Schaaf K. P. Ornato J. P. (2018). Association between ventricular fibrillation amplitude immediately prior to defibrillation and defibrillation success in out-of-hospital cardiac arrest. *Am. Heart J.* 201 72–76. 10.1016/j.ahj.2018.04.002 29910058

[B5] Bradley C. P. Clayton R. H. Nash M. P. Mourad A. Hayward M. Paterson D. J. (2011). Human ventricular fibrillation during global ischemia and reperfusion: paradoxical changes in activation rate and wavefront complexity. *Circulation* 4 684–691. 10.1161/circep.110.961284 21841193

[B6] Brown C. G. Dzwonczyk R. Werman H. A. Hamlin R. L. (1989). Estimating the duration of ventricular fibrillation. *Ann. Emerg. Med.* 18 1181–1185. 10.1016/s0196-0644(89)80056-3 2817561

[B7] Caldwell J. C. Burton F. L. Cobbe S. M. Smith G. L. (2012). Amplitude changes during ventricular fibrillation: a mechanistic insight. *Front. Physiol.* 3:147. 10.3389/fphys.2012.00147 22654763 PMC3358710

[B8] Cheng K. A. Dosdall D. J. Li L. Rogers J. M. Ideker R. E. Huang J. (2012). Evolution of activation patterns during long-duration ventricular fibrillation in pigs. *Am. J. Physiol. Heart Circ. Physiol.* 302 992–1002. 10.1152/ajpheart.00419.2011 22180655 PMC3322740

[B9] Cheniti G. Vlachos K. Meo M. Puyo S. Thompson N. Denis A. (2018). Mapping and ablation of idiopathic ventricular fibrillation. *Front. Cardiovasc. Med.* 5:123. 10.3389/fcvm.2018.00123 30280100 PMC6153961

[B10] Chicote B. Irusta U. Alcaraz R. Rieta J. J. Aramendi E. Isasi I. (2016). Application of entropy-based features to predict defibrillation outcome in cardiac arrest. *Entropy* 18 1–17. 10.3390/e18090313

[B11] Cismaru G. Brembilla-Perrot B. Pauriah M. Zinzius P. Y. Sellal J. M. Schwartz J. (2013). Cycle length characteristics differentiating non-sustained from self-terminating ventricular fibrillation in Brugada syndrome. *Europace* 15 1313–1318. 10.1093/europace/eut023 23419658

[B12] Clayton R. H. Murray A. Campbell R. W. F. (1995). Analysis of the body surface ECG measured in independent leads during ventricular fibrillation in humans. *PACE Pacing Clin. Electrophysiol.* 18 1876–1881. 10.1111/j.1540-8159.1995.tb03835.x 8539155

[B13] De Ambroggi L. Aimè E. Ceriotti C. Rovida M. Negroni S. (1997). Mapping of ventricular repolarization potentials in patients with arrhythmogenic right ventricular dysplasia: principal component analysis of the ST-T waves. *Circulation* 96 4314–4318. 10.1161/01.cir.96.12.4314 9416898

[B14] Di Marco L. Y. Bourke J. P. Langley P. (2012). Spatial complexity and spectral distribution variability of atrial activity in surface ECG recordings of atrial fibrillation. *Med. Biol. Eng. Comput.* 50 439–446. 10.1007/s11517-012-0878-8 22402888

[B15] Eftestøl T. Sunde K. Aase S. O. Husøy J. H. Steen P. A. (2000). Predicting outcome of defibrillation by spectral characterization and nonparametric classification of ventricular fibrillation in patients with out-of-hospital cardiac arrest. *Circulation* 102 1523–1529. 10.1161/01.cir.102.13.1523 11004143

[B16] Fishman G. I. Chugh S. S. Dimarco J. P. Albert C. M. Anderson M. E. Bonow R. O. (2010). Sudden cardiac death prediction and prevention: report from a national heart, lung, and blood institute and heart rhythm society workshop. *Circulation* 122 2335–2348. 10.1161/CIRCULATIONAHA.110.976092 21147730 PMC3016224

[B17] Fitz-Clarke J. R. Sapp J. L. Warren J. W. Clements J. C. Horáček B. M. (2006). Body surface potential mapping and computer simulation of human ventricular fibrillation. *Comput. Cardiol.* 33 397–400.

[B18] Ganesan A. N. Kuklik P. Lau D. H. Brooks A. G. Baumert M. Lim W. W. (2013). Bipolar electrogram Shannon entropy at sites of rotational activation implications for ablation of atrial fibrillation. *Circulation* 6 48–57. 10.1161/circep.112.976654 23264437

[B19] Gray R. A. Pertsov A. M. Jalife J. (1998). Spatial and temporal organization during cardiac fibrillation. *Nature* 392 75–78. 10.1038/32164 9510249

[B20] Haïssaguerre M. Hocini M. Cheniti G. Duchateau J. Sacher F. Puyo S. (2018). Localized structural alterations underlying a subset of unexplained sudden cardiac death. *Circulation* 11:e006120. 10.1161/CIRCEP.117.006120 30002064 PMC7661047

[B21] Hajeb-Mohammadalipour S. Ahmadi M. Shahghadami R. Chon K. H. (2018). Automated method for discrimination of arrhythmias using time, frequency, and nonlinear features of electrocardiogram signals. *Sensors* 18:2090. 10.3390/s18072090 29966276 PMC6068712

[B22] Hayashi M. Shimizu W. Albert C. M. (2015). The spectrum of epidemiology underlying sudden cardiac death. *Circ. Res.* 116 1887–1906. 10.1161/circresaha.116.304521 26044246 PMC4929621

[B23] Jalife J. (2000). Ventricular fibrillation: mechanisms of initiation and maintenance. *Annu. Rev. Physiol.* 62 25–50. 10.1146/annurev.physiol.62.1.25 10845083

[B24] Mäkikallio T. H. Huikuri H. V. Myerburg R. J. Seppänen T. Kloosterman M. Interian A. (2002). Differences in the activation patterns between sustained and self-terminating episodes of human ventricular fibrillation. *Ann. Med.* 34 130–135. 10.1080/07853890252953527 12108576

[B25] Malik M. Acar B. Gang Y. I. Yap Y. G. Hnatkova K. John Camm A. (2000). QT dispersion does not represent electrocardiographic interlead heterogeneity of ventricular repolarization. *J. Cardiovasc. Electrophysiol.* 11 835–843. 10.1111/j.1540-8167.2000.tb00061.x 10969744

[B26] Meo M. Bear L. R. Abell E. Cluitmans M. Jaïs P. Hocini M. (2020). *Noninvasive Tracking of Repolarization Gradients as a Substrate for Ventricular Fibrillation.* San Diego, CA: Heart Rhythm Society Congress, 383–476.

[B27] Meo M. Pambrun T. Derval N. Dumas-Pomier C. Puyo S. Duchâteau J. (2018). Noninvasive assessment of atrial fibrillation complexity in relation to ablation characteristics and outcome. *Front. Physiol.* 9:929. 10.3389/fphys.2018.00929 30065663 PMC6056813

[B28] Meo M. Potse M. Puyo S. Bear L. Hocini M. Häissaguerre M. (2017). “Non-invasive assessment of spatiotemporal organization of ventricular fibrillation through principal component analysis,” in *Proceedings of the Computing in Cardiology* (Rennes: IEEE). 10.22489/CinC.2017.101-051

[B29] Meo M. Zarzoso V. Meste O. Latcu D. G. Saoudi N. (2013a). Catheter ablation outcome prediction in persistent atrial fibrillation using weighted principal component analysis. *Biomed. Signal Process. Control.* 8 958–968. 10.1016/j.bspc.2013.02.002

[B30] Meo M. Zarzoso V. Meste O. Latcu D. G. Saoudi N. (2013b). Spatial variability of the 12-lead surface ECG as a tool for noninvasive prediction of catheter ablation outcome in persistent atrial fibrillation. *IEEE Trans. Biomed. Eng.* 60 20–27. 10.1109/tbme.2012.2220639 23033326

[B31] Moe G. K. Abildskov J. Han J. (1964). *Factors Responsible for the Initiation and Maintenance of Ventricular Fibrillation. Sudden Cardiac Death.* New York, NY: Grune & Stratton.

[B32] Muñoz J. J. S. Álvarez J. L. R. Alberola A. G. Carrión J. R. Everss E. Ortiz M. (2009). Spectral analysis of sustained and non-sustained ventricular fibrillation in patients with an implantable cardioverter-defibrillator. *Rev. Española Cardiol.* 62 690–693. 10.1016/S1885-5857(09)72234-0 19480766

[B33] Ng J. Goldberger J. J. (2014). The ups and downs of ventricular fibrillation waveforms. *J. Am. College Cardiol.* 64 1370–1372. 10.1016/j.jacc.2014.07.953 25257640

[B34] Oh S. L. Hagiwara Y. Adam M. Sudarshan V. K. Koh J. E. Tan J. H. (2017). Shockable versus nonshockable life-threatening ventricular arrhythmias using dwt and nonlinear features of ECG signals. *J. Mech. Med. Biol.* 17:1740004 10.1142/s0219519417400048

[B35] Park S. A. Gray R. A. (2015). Optical mapping of ventricular fibrillation dynamics. *Membr. Potential Imaging Nerv. Syst. Heart* 313–342. 10.1007/978-3-319-17641-3_13 26238059

[B36] Patwardhan A. Moghe S. Wang K. E. Leonelli F. (2000). Frequency modulation within electrocardiograms during ventricular fibrillation. *Am. J. Physiol. Heart Circ. Physiol.* 279 825–835. 10.1152/ajpheart.2000.279.2.H825 10924083

[B37] Peters S. Peters H. Thierfelder L. (1999). Risk stratification of sudden cardiac death and malignant ventricular arrhythmias in right ventricular dysplasia-cardiomyopathy. *Int. J. Cardiol.* 71 243–250. 10.1016/s0167-5273(99)00142-4 10636530

[B38] Priori S. G. Blomström-Lundqvist C. Mazzanti A. Blom N. Borggrefe M. Camm J. (2015). ESC Guidelines for the management of patients with ventricular arrhythmias and the prevention of sudden cardiac death. *Eur. Heart J.* 36 2793–2867. 26745817 10.1093/eurheartj/ehv445

[B39] Priori S. G. Mortara D. W. Napolitano C. Diehl L. Paganini V. Cantù F. (1997). Evaluation of the spatial aspects of T-wave complexity in the long-QT syndrome. *Circulation* 96 3006–3012. 10.1161/01.cir.96.9.3006 9386169

[B40] Robson J. Aram P. Nash M. P. Bradley C. P. Hayward M. Paterson D. J. (2018). Spatio-temporal organization during ventricular fibrillation in the human heart. *Ann. Biomed. Eng.* 46 864–876. 10.1007/s10439-018-2007-9 29546467 PMC5934463

[B41] Rogers J. M. Huang J. Smith W. M. Ideker R. E. (1999). Incidence, evolution, and spatial distribution of functional reentry during ventricular fibrillation in pigs. *Circ. Res.* 84 945–954. 10.1161/01.res.84.8.945 10222342

[B42] Sörnmo L. Laguna P. (2005). *Bioelectrical Signal Processing in Cardiac and Neurological Applications.* Amsterdam: Elsevier.

[B43] Steg P. G. James S. K. Atar D. Badano L. P. Lundqvist C. B. Borger M. A. (2012). ESC Guidelines for the management of acute myocardial infarction in patients presenting with ST-segment elevation. *Eur. Heart J.* 33 2569–2619. 22922416 10.1093/eurheartj/ehs215

[B44] Strohmenger H. U. Eftestol T. Sunde K. Wenzel V. Mair M. Ulmer H. (2001). The predictive value of ventricular fibrillation electrocardiogram signal frequency and amplitude variables in patients with out-of-hospital cardiac arrest. *Anesth. Analgesia* 93 1428–1433. 10.1097/00000539-200112000-00016 11726418

[B45] Such-Miquel L. Chorro F. J. Guerrero J. Trapero I. Brines L. Zarzoso M. (2013). Evaluation of the complexity of myocardial activation during ventricular fibrillation. An experimental study. *Rev. Esp. Cardiol.* 66 177–184. 10.1016/j.rec.2012.08.012 24775451

[B46] Tovar O. H. Jones J. L. (2000). Electrophysiological deterioration during long-duration ventricular fibrillation. *Circulation* 102 2886–2891. 10.1161/01.cir.102.23.2886 11104749

[B47] Turrini P. Corrado D. Basso C. Nava A. Bauce B. Thiene G. (2001). Dispersion of ventricular depolarization-repolarization. *Circulation* 103 3075–3080. 10.1161/01.cir.103.25.3075 11425771

[B48] Umapathy K. Nair K. Masse S. Krishnan S. Rogers J. Nash M. P. (2010). Phase mapping of cardiac fibrillation. *Circulation* 3 105–114. 10.1161/CIRCEP.110.853804 20160178

[B49] Weiss J. N. Qu Z. Chen P. S. Lin S. F. Karagueuzian H. S. Hayashi H. (2005). The dynamics of cardiac fibrillation. *Circulation* 112 1232–1240. 16116073 10.1161/CIRCULATIONAHA.104.529545

[B50] Winkle R. A. Mead R. H. Ruder M. A. Smith N. A. Buch W. S. Gaudiani V. A. (1990). Effect of duration of ventricular fibrillation on defibrillation efficacy in humans. *Circulation* 81 1477–1481. 10.1161/01.cir.81.5.1477 2331763

[B51] Wltkowskl F. X. Leon L. J. Penkoske P. A. Giles W. R. Spano M. L. Dltto W. L. (1998). Spatiotemporal evolution of ventricular fibrillation. *Nature* 392 78–82. 10.1038/32170 9510250

[B52] Zhang F. Hamon D. Fang Z. Xu Y. Yang B. Ju W. (2017). Value of a posterior electrocardiographic lead for localization of ventricular outflow tract arrhythmias: the V4/V8 ratio. *JACC Clin. Electrophysiol.* 3 678–686. 10.1016/j.jacep.2016.12.018 29759536

